# Antioxidant and Antimicrobial Effects of Baby Leaves of *Amaranthus tricolor* L. Harvested as Vegetable in Correlation with Their Phytochemical Composition

**DOI:** 10.3390/molecules28031463

**Published:** 2023-02-02

**Authors:** Aneta Spórna-Kucab, Anna Tekieli, Aneta Kisiel, Agnieszka Grzegorczyk, Krystyna Skalicka-Woźniak, Karolina Starzak, Sławomir Wybraniec

**Affiliations:** 1 Department of Chemical Technology and Environmental Analytics, Faculty of Chemical Engineering and Technology, Cracow University of Technology, Warszawska 24, 31-155 Kraków, Poland; 2 Chair and Department of Pharmaceutical Microbiology, Medical University of Lublin, 1 Chodźki Str., 20-093 Lublin, Poland; 3 Department of Natural Products Chemistry, Medical University of Lublin, 1 Chodźki Str., 20-093 Lublin, Poland

**Keywords:** *amaranthus*, antimicrobial, antioxidant, correlation, HRMS

## Abstract

Amaranth is used as a spinach replacement; therefore, it is sometimes called Chinese Spinach. So far, the activity of the plant has not been associated with the presence of specific compounds. Three cultivars of *Amaranthus tricolor* L. were investigated for their antioxidant and antimicrobial activities. The correlation between the bioactivity and metabolite profiles was investigated in order to indicate active compounds in *A. tricolor*. The phytochemical profile of a total of nine extracts was studied by HPLC-DAD-ESI/HRMS, revealing the presence of 52 compounds. The highest antioxidant activity was noticed in the Red cultivar (0.06 mmol TE/g DE (Trolox Equivalent/Dry Extract Weight) and was related to the presence of amino acids, flavonoids and phenolic acids, as well as individual compounds such as tuberonic acid hexoside. All studied extracts revealed antimicrobial activity. Gram-positive bacteria were more susceptible to *N*-(carboxyacetyl) phenylalanine, phenylalanine, tuberonic acid and succinic acid and Gram-negative bacteria to dopa, tryptophan, norleucine, tuberonic acid hexoside, quercetin-*O*-hexoside, luteolin-*O*-rhamnosylhexoside, luteolin-6-*C*-hexoside succinic acid, gallic acid-*O*-hexoside, dihydroxybenzoic acid and hydroxybenzoic acid. Maleic acid showed promising antifungal activity. In summary, *A. tricolor* is a good source of antioxidant and antimicrobial compounds.

## 1. Introduction

Amaranth is a well-known and popular plant. In Central America, it was the main food source along with corn and beans. Leaves, stems and seeds may be eaten raw and cooked. The leaves have a high nutritional value. It is one of the easiest plants to cultivate due to its resistance to environmental conditions. There are over 800 varieties of *Amaranthus*, and among them is *Amaranthus tricolor* L. [1,2,3], which have been used as a traditional medicine in Siddha and Ayurveda in the treatment of diarrhea, menorrhagia, intestinal hemorrhage, dysentery, hemorrhagic colitis, bronchitis and cough [4]. *A. tricolor* is used in the treatment of a number of diseases. Studies have shown that crude extracts of *A. tricolor* leaves have antioxidant, hepatoprotective, antimicrobial, anti-inflammatory and anticancer activities [1,5].

There has been an increase in interest in the use of compounds that prevent or reduce the effects of oxidative stress (OS) in living cells [5,6,7]. OS plays an important role in the aging process and the development of chronic and degenerative diseases [7]. Antioxidants have different mechanisms of action, as well as solubility, redox potential and mechanisms of action. They can quench reactive oxygen species (ROS) and reactive nitrogen species (RNS). It is possible that others may interfere with the oxidizing of metal ions or inhibit the activity of oxidative enzymes. The presence of antioxidants may increase the potential of other compounds [6]. The study of the antioxidant potential of compounds is carried out using various methods taking into account different reaction mechanisms [6,7].

The antimicrobial activity of the compounds may be related to their antioxidant properties. Microbial invasion can lead to inflammatory processes in the body with the release of a large variety of oxidants, cytokines and proteolytic enzymes by various cells of the immune system. Insufficient responses from the immune system will lead to elevated microbial colonization. Compounds with antimicrobial activities have been shown to be more effective in reducing the immune system responses [6].

There are studies of *A. tricolor* leaves (cv. Valentina). A total of 41 metabolites were indicated, including amino acids, organic acids, phenolic acids and fatty acids [8,9]. *A. tricolor* is a source of a rare group of highly bioactive compounds—betacyanins [10,11], which are not common in nature. Their occurrence is limited to a few families of the Caryophyllales order and some higher-order fungi. There is significant interest in these compounds due to their confirmed antioxidant, anti-inflammatory, antimicrobial, anticancer, neuroprotective and hepatoprotective activities [1,5].

The activity of *A. tricolor* has not been associated with specific compounds. The study reports correlations between the antimicrobial, antioxidant and phytochemical profiles of various edible *A. tricolor* Calaloo cv. Red, Passion and Green. Fresh leaves used as leafy vegetables were extracted in food-acceptable solvents: water, ethanol and acetone. Furthermore, the study of the phytochemical profile indicates the best source of active compounds (variety and extraction conditions). Research on the metabolites, as well as antioxidant and antimicrobial properties of *A. tricolor* Calaloo cultivars, were performed for the first time.

## 2. Results and Discussion

### 2.1. HPLC-DAD-ESI/HRMS Metabolite Profile

The HPLC-DAD-ESI/HRMS analysis of the nine extracts of *A. tricolor* Calaloo cultivars revealed the presence of 52 compounds, including 12 amino acids, 4 betacyanins, 9 fatty acids, 5 flavonoids, 8 organic acids and 14 phenolic acids (Section 2.1.1, Section 2.1.2, Section 2.1.3, Section 2.1.4, Section 2.1.5 and Section 2.1.6).

The tentative structural identification of the described metabolites is based on a multidimensional analytical approach consisting of comparing the profiles of identified compounds in amaranth, the exact mass of the identified structures and MS/MS fragments with MS data with the previous literature data reported for amaranthus [4,9,12] and other plants [13,14,15,16,17,18,19,20,21,22,23,24,25,26] or online databases (PubChem, PhytoHub, MoNA and HMDB).

#### 2.1.1. Amino Acids

**Figure 1:**
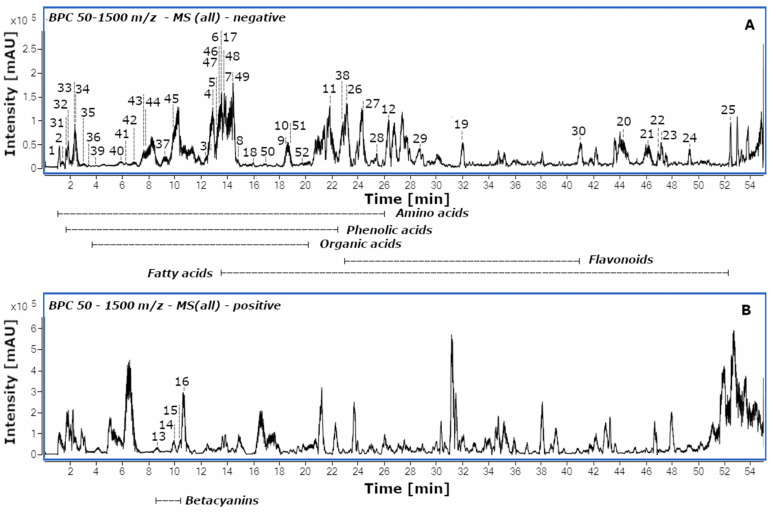
Base peak chromatograms (BPC) of the Red variety of *A. tricolor* extracted with water obtained by high-performance diode array detector-electrospray ionization/high-resolution mass spectrometry (HPLC-DAD-ESI/HRMS) in negative (**A**) and positive (**B**) ion modes. The compound numbers and names are described in Table 1.

Twelve amino acids were tentatively identified (Figure 1; Table 1) on the basis of their HRMS, MS/MS data and comparison with the fragmentation pathways described in the literature [13,14,15,16,17]. Among the detected compounds, serine (**1**, *m*/*z* 104.0356), aspartic acid (**2**, *m*/*z* 132.0301), *N*-benzoylaspartic acid (**4**, *m*/*z* 236.0564) and tryptophan (**11**, *m*/*z* 203.0826) were dominant in *A. tricolor* cv. Calaloo.

The eight amino acids, except *N*-benzoylaspartic acid (**4**), *N*-(carboxyacetyl) phenylalanine (**7**, *m*/*z* 250.0720), dopa (**8**, *m*/*z* 196.0624) and norleucine (**12**, *m*/*z* 130.0873), were identified previously in the *Amaranthus* L. genus [27].

The MS/MS spectrum provided fragments obtained by losing the CO_2_ group (−44 Da) and/or NH_3_ (−17 Da) from the molecule, which is characteristic of amino acids. Serine (**1**), aspartic acid (**2**), glutamic acid (**5**, *m*/*z* 146.0460) and *N*-(carboxyacetyl) phenylalanine (**7**, *m*/*z* 250.0720) showed important fragment ions at *m*/*z* 60.0441, 88.0422, 102.0559 and 206.0824, which correspond to the loss of CO_2_.

Serine was previously identified in *A. tricolor* cv. Valentina [9]. The loss of CO_2_ and NH_3_ (ammonia) was noticed in tyrosine (**3**, *m*/*z* 180.0662), isoleucine (**6**, *m*/*z* 130.0875), dopa (**8**, *m*/*z* 196.0624), leucine (**9**, *m*/*z* 130.0880), tryptophan (**11**) and norleucine (**12**, *m*/*z* 130.0873). Isoleucine (**6**), leucine (**9**) and tryptophan (**11**) were reported in *A. caudatus* [29]. Phenylalanine (**10**, *m*/*z* 164.0710) showed an important ion at *m*/*z* 147.0432, which corresponded to the loss of the ammonia group. *N*-benzoylaspartic acid (**4**, *m*/*z* 236.0564) fragmentation generated ions at *m*/*z* 192.0695 and 174.9560163 due to the loss of CO_2_ and H_2_O, respectively (Figure 1; Table 1).

#### 2.1.2. Betacyanins

The *Amaranthus* genus has been reported as a very good source of amaranthin and isoamaranthin, but there is no detailed report on the betacyanin profile in edible leaves of young *A. tricolor* cv. Callaloo. Extensive research in the genus *Amaranthus* L. demonstrated that amaranthin and isoamaranthin are dominant compounds. The authors also indicate the presence of betanin/isobetanin, celosianin I/isocelosianin I and celosianin II/isocelosianin II in selected species [10].

**Figure 2:**
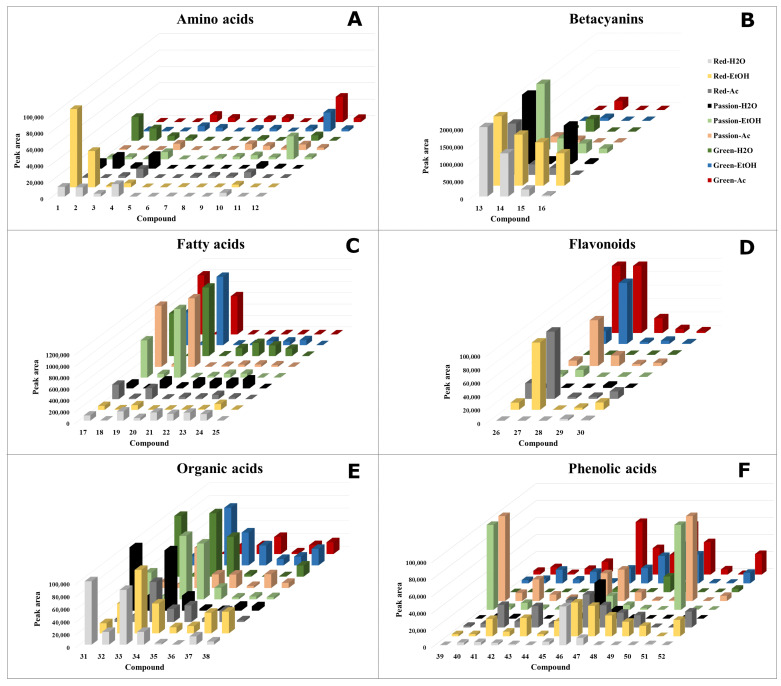
Absolute peak areas of identified metabolites of the Red, Green and Passion variety of *A. tricolor* extracted with water, ethanol and acetone obtained by high-performance-diode array detector-electrospray ionization/high-resolution mass spectrometry (HPLC-DAD-ESI/HRMS) (**A**–**F**). The compound numbers and names are described in Table 1.

Here, we present four betacyanins identified by comparison with authentic standards isolated and described in previous experiments [11]: amaranthin (**13**, *m*/*z* 727.1826), betanin (**14**, *m*/*z* 551.1508), isoamaranthin (**15**, *m*/*z* 727.1832) and isobetanin (**16**, *m*/*z* 551.1516). Amaranthin (**13**) and isoamaranthin (**15**), as well as betanin (**14**) and isobetanin (**16**), are isomers with an identical degradation pathway leading to obtaining an aglycone—betanidin. Betanidin and isobetanidin have been reported for the first time in the *Amaranthus* L. genus. Here, amaranthin (**13**) was the dominant compound in *A. tricolor* cv. Calaloo. The highest content of all betacyanins was noticed in Red-EtOH, whereas the lowest content was observed for Green-H_2_O/EtOH/Ac (Figure 1 and Figure 2; Table 1).

#### 2.1.3. Fatty Acids

Nine fatty acids were tentatively identified (Figure 1 and Figure 2; Table 1), including two jasmonate derivatives: tuberonic acid (**18**, *m*/*z* 225.1132) and its conjugate tuberonic acid hexoside (**17**, *m*/*z* 387.1673), as well as derivatives of octadecadienoic and octadecatrienoic acids (**19**–**25**). The fragmentation of **17** generated ions at *m*/*z* 225.1132, 207.0976 and 163.0400 due to the sequential loss of the glucose moiety (−162 Da), H_2_O (−18 Da) and CO_2_ (−44 Da). Compound **18** showed a fragment ion at *m*/*z* 163.0425, which corresponds to the loss of H_2_O (−18 Da) and CO_2_ (−44 Da) [20]. The octadecadienoic acid is substituted with one (279 + 16 = 295 Da) or a trihydroxy moiety (279 + 48 = 327 Da). Compounds **24** (*m*/*z* 295.2279), **25** (*m*/*z* 295.2280) and **19** (*m*/*z* 327.2181) were identified as hydroxy and trihydroxyoctadecadienoic acids. Compound **20** (*m*/*z* 311.2233) was proposed as hydroperoxyoctadecadienoic acid, according to the MS information (279 + 32 = 311 Da). Octadecatrienoic acid was substituted with one hydroxy moiety (277 + 16 = 293 Da). Three hydroxyoctadecatrienoic acids (**21**–**23**) were detected. The MS/MS spectrum of hydroxyoctadecatrienoic and hydroxyoctadecadienoic acids provided fragments obtained by losing H_2_O (−18 Da) from the structure, which is characteristic for hydroxy fatty acids [30]. Fatty acids were previously identified in the genus *Amaranthus* [9] but solely palmitic acid, stearic acid, oleic acid and linoleic acid. Here, the fatty acids were mainly extracted from *A. tricolor* Calaloo Passion and Green. Furthermore, tuberonic acid hexoside (**17**) and trihydroxyoctadecadienoic acid (**19**) were the dominant compounds. 

#### 2.1.4. Flavonoids

Flavonoids were the least numerous group of compounds identified. Five derivatives were noted—among them, quercetin-*O*-hexoside (**26**, *m*/*z* 463.0886) and luteolin-*O*-rhamnosylhexoside (**27**, *m*/*z* 593.1514), which were the dominant compounds (Figure 1 and Figure 2; Table 1). These compounds extracted mainly with ethanol and acetone proved the MS/MS spectral data similar to those from Gök et al. [19].

Glucoside degradation is usually based on the deglycosylation reaction [11,18]; therefore, we noticed fragments corresponding to aglycones: quercetin (*m*/*z* 301.0333) and luteolin (*m*/*z* 285.0180). The loss of 162 Da (−glucose) was also observed in luteolin-6-*C*-hexoside (**28**, *m*/*z* 447.0944), phlorizin (**29**, *m*/*z* 435.1301) and luteolin-7-*O*-glucoside (**30**, *m*/*z* 447.0933). The MS/MS spectra showed fragment ions at *m*/*z* 285.1535, 273.1210 and 285.0174, respectively. Similar ion fragments were noticed in data published by Zengin et al. [21]. Quercetin glycosides were previously identified in *Amaranthus* L. genus [12].

#### 2.1.5. Organic Acids

Eight compounds were characterized as organic acids. Compound **31** (*m*/*z* 191.0199) was identified as citric acid, for which the fragment was at *m*/*z* 111.0072 due to the loss of H_2_O and CO_2_ [23]. Mannonic acid (**32**, *m*/*z* 195.0510), malic acid (**33**, *m*/*z* 133.0134) and glyceric acid (**36**, *m*/*z* 105.0192) showed fragment ions correspond to the loss of H_2_O, whereas the loss of CO_2_ was noticed in maleic acid (**34**, *m*/*z* 115.0037), succinic acid (**35**, *m*/*z* 117.0193), 2-oxopentanoic acid (**37**, *m*/*z* 115.0402) and fumaric acid (**38**, *m*/*z* 115.0037). These organic acids, except citric acid and malic acid, were previously described in *A. tricolor* [9]. Here, citric acid (**31**), mannonic acid (**32**), malic acid (**33**) and maleic acid (**34**) are dominant in *A. tricolor* cv. Calaloo (Figure 1 and Figure 2; Table 1).

#### 2.1.6. Phenolic Acids

Phenolic acids were the most abundant constituents of the three species of *A*. *tricolor*. Performed on the basis of HRMS, MS/MS data and comparison with fragmentation paths described in the literature [15,17,19,21,25,26], 14 compounds were tentatively described (Figure 1 and Figure 2; Table 1). 

The MS/MS spectrum provided fragments obtained by losing the CO_2_ group (−44 Da) from the molecule, which is characteristic for phenolic acids. This regularity was noticed for both phenolic acids and glycosylated conjugates [19]. 

Gallic acid (**39**, *m*/*z* 169.0152) and vanillic acid (**42**, *m*/*z* 167.0345) were identified with their glycosylated derivatives: gallic acid-*O*-hexoside **40** (169 + 162 = 331 Da) and vanillic acid-*O*-hexoside (**41**) (167 + 162 = 329 Da). Both gallic (**39**) acid and the glucosylated conjugate showed specific MS fragments at *m*/*z* 125.0247 due to the loss of the CO_2_ group. Similarly, for vanillic acid and its glucoside, the characteristic fragment at *m*/*z* 123.0442 was noticed. Protocatechuic acid-*O*-hexoside (**43**, *m*/*z* 315.0729) was detected, and its MS/MS spectra showed fragments at *m*/*z* 153.0197 and 109.0304, corresponding to the loss of 162 Da (-glucose) and 44 Da (−CO_2_). Benzoic acid (**46**, *m*/*z* 121.0298), substituted with the hydroxy moiety, compounds **44** (*m*/*z* 153.0195) and **45** (*m*/*z* 137.0244) were proposed as dihydroxybenzoic acid (121 + 32 = 153 Da) and hydroxybenzoic acid (121 + 16 = 137 Da). The characteristic fragment at *m*/*z* 77.1045 showed the loss of the CO_2_ (−44 Da) from benzoic acid.

Based on previous HRMS, MS/MS data [15,21,25], ferulic acid (**47**, *m*/*z* 193.0503), *p*-coumaric acid (**48**, *m*/*z* 163.0401) and *o*-coumaric acid (**49**, *m*/*z* 193.0503) were identified. Fragment ions at *m*/*z* 197.0457, 147.0452 and 179.0350 were putatively annotated as syringic acid (**50**), cinnamic acid (**51**) and caffeic acid (**52**). Fragments at *m*/*z* 153.1034, 103.0530 and 135.0430 were observed due to the loss of the CO_2_ group. 

The highest contents of the phenolic acids, gallic acid (**39**) and syringic acid (**50**) were noticed in Passion-EtOH/Ac. Benzoic acid (**46**), ferulic acid (**47**), *p*-coumaric acid (**48**) and *o*-coumaric acid (**49**) were also identified with high contents in *A. tricolor* cv. Calaloo (Figure 1 and Figure 2; Table 1).

### 2.2. Antioxidant Assays

**Figure 3:**
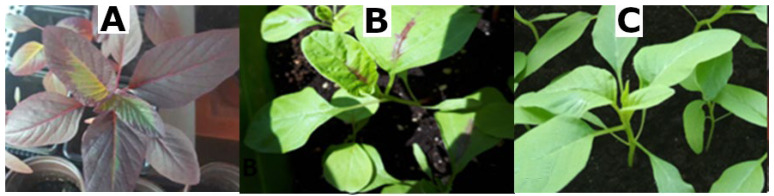
Three cultivars of *A. tricolor* Calaloo: Red (**A**), Passion (**B**) and Green (**C**).

**Figure 4:**
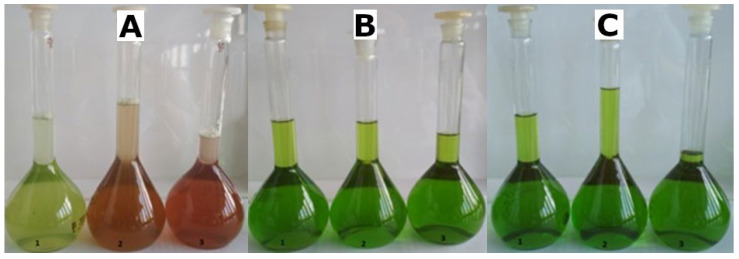
Green (**1**), Passion (**2**) and Red (**3**) varieties of *A*. *tricolor* Calaloo extracted with water (**A**), ethanol (**B**) and acetone (**C**).

The antioxidative capacities of the H_2_O/EtOH/Ac extracts of three *A. tricolor* Calaloo cultivars: Red, Passion and Green (Figure 3 and Figure 4) were assessed by employing three in vitro cell-free assays: ABTS, FRAP and DPPH, and the results are shown in Table 2 expressed as mmol Trolox Equivalents per grams of dry extract weight (DE).

In the ABTS assay, the values were in the range of 0.015 to 0.060 mmol TE/g DE, which represents a variation 4-fold. In this test, Red-H_2_O showed the highest antioxidant activity (0.060 mmol TE/g DE) compared to the other varieties, while the lowest activity showed Green-Ac (0.015 mmol TE/g DE).

In the DPPH assay, the values were in the range of 0.009 to 0.021 mmol TE/g DE, which represents a variation of approximately 2-fold. In this assay, Red-H_2_O also possessed the highest antioxidant activity (0.021 mmol TE/g DE), while the lowest activity also showed Green-Ac (0.009 mmol TE/g DE).

The FRAP values varied from 0.009 to 0.053 mmol TE/g DE, which represents a higher variation than in the ABTS and DPPH assays of 6-fold. In this test, Red-H_2_O also had the highest antioxidant activity (0.053 mmol TE/g DE). Green-Ac possessed the lowest antioxidant potential (0.009 mmol TE/g DE) for the ABTS and DPPH tests.

The extrahent had a great influence on the antioxidant activity, which can be seen in Table 2. According to the three ABTS, FRAP and DPPH tests used within a given variety of *A. tricolor*, the activity decreased depending on the solvent used in the sequence water > ethanol> acetone.

Ascorbic acid is very popular for its antioxidant properties, so it was used as a reference compound. The investigated extracts of three *A*. *tricolor* Calaloo cultivars (Red, Passion and Green) showed much lower antioxidant activity than pure ascorbic acid. However, it is important to remember that the plant material matrix contains a wide range of different compounds, possibly including those that can inhibit free radical scavenging. Therefore, comparisons of the results for pure compounds with the results obtained on complex extracts should be treated with approximation. 

The obtained results of the antioxidant activity of ascorbic acid measured by the ABTS and DPPH methods are similar (7.8 and 8.1 mmol TE/g DE, respectively). However, the result obtained by the FRAP method (4.6 mmol TE/g DE) differs significantly from the others, which may be due to the fact that the presence of iron (III) ions in the FRAP reagent can significantly intensify the oxidation of ascorbic acid [31].

#### Correlation between Antioxidant Activity and Phytochemical Composition

The antioxidant activity results (mmol TE/g DE) of the *A*. *tricolor* Calaloo extracts were correlated with their metabolite composition (absolute peak area of each assigned peak from the chromatograms) and are presented in Table 2.

There was no significant correlation between the antioxidant activity and betacyanins or organic acids (*p* <0.05), which clearly shows that other compounds might be responsible for the antioxidant potential of the extracts tested. However, the antioxidant potential of individual, pure betacyanins and organic acids cannot be excluded, which has been confirmed previously in numerous times in the literature [10,11,32,33]. It should be noted that Zhang et al. [34] also investigated the correlation between organic acids and antioxidant activity measured by the three DPPH, ABTS and FRAP assays. These results indicate that organic acids exhibited a very low contribution to the total antioxidant activity [34].

Our experiments revealed that three compounds from the group of amino acids (glutamic acid (**5**), dopa (**8**) and norleucine (**12**) showed a strong positive correlation (R = 0.5-0.7). Dopa had a significant and strong correlation with the antioxidant activity measured by the ABTS, FRAP and DPPH methods (R = 0.550, 0.690 and 0.640, respectively). The glutamic acid correlations shown with the FRAP and DPPH assays were R = 0.539 and 0.524, respectively; however, norleucine had a significant and strong correlation assessed only by the FRAP method (R = 0.595). 

It has been confirmed in the literature that glutamic acid markedly increases the total phenol concentration and antioxidant activity [35]. Dopa, used as a drug in Parkinson’s disease, has been found to be an effective antioxidant in different in vitro assays, including anti-lipid peroxidation; reductive ability; ABTS, DPPH and superoxide anion radical scavenging activities; hydrogen peroxide scavenging and metal chelating activities with efficiency compared to the standard antioxidant compounds, such as *α*-tocopherol and Trolox. Furthermore, Dopa oxidation products prevented H_2_O_2_-induced oxidative damage to cellular DNA in cultured tissue cells [36]. Norleucine was also confirmed to have unusually strong antioxidant activity [37]. Here, glutamic acid (**5**), dopa (**8**) and norleucine (**12**) were not dominant amino acids in the *A. tricolor* Calaloo varieties. A higher content of these compounds was noticed in *A. tricolor* Calaloo Passion and Green.

Almost all fatty acids showed negative or weak correlations (R = 0-0.3) with the antioxidant activity. An exception to this was tuberonic acid hexoside (**17**), which showed a very strong (R = 0.7–1.0) and significant correlation with the antioxidant potential evaluated by the FRAP assay (R = 0.755) and a strong and significant correlation (R = 0.5–0.7) with the activity evaluated by the DPPH assay (R = 0.678). *A. tricolor* Calaloo seems to be a rich source of tuberonic acid hexoside (**17**). Trihydroxyoctadecadienoic acid (**19**) present in high concentrations in *A. tricolor* also showed correlations with the antioxidant potential assessed by the FRAP (R = 0.068) and the DPPH (R = 0.239) assays. 

The oxidation fatty acids rate depends on the number of double bonds in the carbon chain. Therefore, the susceptibility to oxidation increases exponentially in proportion to the number of unsaturated bonds in fatty acids. Numerous studies have confirmed the antioxidant activity of fatty acids [38,39,40].

Almost all compounds from the flavonoids group showed a positive correlation, while only luteolin-6-*C*-hexoside (**28**) had significant and very strong correlations with the antioxidant activity measured by the ABTS, FRAP and DPPH assays (R = 0.765, 0.885 and 0.703, respectively), which the literature confirmed [12,21]. It is worth noting that quercetin *O*-hexoside (**26**) showed very strong and significant correlation with the antioxidant potential evaluated only by the FRAP method (R = 0.775) and a strong correlation against activity assessed by the ABTS and DPPH methods (R = 0.590 and 0.500, respectively). Both compounds are present in high concentrations in *A. tricolor* extracts.

Compounds belonging to the phenolic acid group showed both positive and negative correlations. Only hydroxybenzoic acid (**45**) had very strong and significant correlations with the antioxidant potential assessed by the ABTS and FRAP assays (R = 0.708 and 0.772, respectively) and a strong correlation with activity evaluated only by the DPPH method (R = 0.546). Gallic acid (**39**) showed strong and significant correlations with activity assessed by the methods ABTS, FRAP and DPPH (R = 0.629, 0.649 and 0.541, respectively). However, three compounds from this group (vanillic acid (**42**), dihydroxybenzoic acid (**44**) and *p*-coumaric acid (**48**) showed a strong correlation with the antioxidant potential assessed by only one assay.

In the past decade, phenolic acids have been demonstrated to possess potent antioxidant activities, which mainly depend on the number and arrangement of hydroxyl groups. In addition, they are indicated as antimicrobials.

### 2.3. Antimicrobial Activity

The antimicrobial activity of three varieties of *A. tricolor* leaves Calaloo: Red, Passion and Green-H_2_O/EtOH/Ac was tested using the broth microdilution method and characterized by the parameters MIC, MBC and MFC defining the minimum inhibitory concentrations of the antimicrobial agent in the respective strains of bacteria or yeast. 

The extracts showed differential activity against Gram-positive bacteria (MIC = 4-32 mg/mL), Gram-negative bacteria (MIC = 4–32 mg/mL) and yeasts (MIC = 8–32 mg/mL), indicating their different health effects. To assess the bactericidal/fungicidal (MBC/MIC ≤ 4, MFC/MIC ≤ 4) or the bacteriostatic/fungistatic (MBC/MIC > 4, MFC/MIC > 4) effect, the extracts tested used MBC/MIC or MFC/MIC ratios. 

The highest antimicrobial activity was observed for Red-Ac and Green-Ac with bactericidal, fungicidal and bacteriostatic effects (without Calaloo Green). Red-Ac showed the highest activity with a bactericidal effect (MBC/MIC ≤ 4) against strains of Gram-positive bacteria *B. cereus* ATCC 10876 (MIC = 4 mg/mL) and Gram-negative bacteria *B. bronchiseptica* ATCC 4617 (MIC = 4 mg/mL) and very good activity with bactericidal effect (MBC/MIC ≤ 4) against three strains of Gram-positive bacteria: *S. aureus* ATCC 25923, *B. subtilis* ATCC 6633 and *M. luteus* ATCC 10240 (for all three strains, MIC = 8 mg/mL) and very good activity with a fungicidal effect (MFC/MIC ≤ 4) against yeast strain *C. krusei* ATCC 14243 (MIC = 8 mg/mL), as well as the highest activity with a bacteriostatic effect (MBC/MIC > 4) against strains of Gram-positive bacteria *S. aureus* ATCC 6538 and *S. aureus* ATCC 29213 (for both strains, MIC = 4 mg/mL). 

Green-Ac showed the highest activity with a bactericidal effect (MBC/MIC ≤ 4) against six strains of Gram-positive bacteria: *S. aureus* ATCC 25923, * B. subtilis* ATCC 6633, *S. aureus* ATCC 6538, *S. epidermidis* ATCC 12228, *B. cereus* ATCC 10876 and *S. aureus* ATCC BAA1707 (for all strains, MIC = 8 mg/mL) and against a strain of Gram-negative bacterial: *P. aeruginosa* ATCC 27853 (MIC = 8 mg/mL) and activity with a fungicidal effect (MFC/MIC ≤ 4) against *C. albicans* ATCC 10231 and *C. glabrata* ATCC 90030 (for both strains MIC = 8 mg/mL). 

Red-EtOH and Green-H_2_O/EtOH showed identical activity with a bactericidal effect (MBC/MIC ≤ 4) against the strain of Gram-positive bacterial *M. luteus* ATCC 10240 (MIC = 8 mg/mL) and for two strains of Gram-negative bacteria: *P. aeruginosa* ATCC 27853 (MIC = 8 mg/mL) and *B. bronchiseptica* ATCC 4617 (MIC = 4 mg/mL), as well as a fungicidal effect (MFC/MIC ≤ 4) against three strains of yeast *C. glabrata* ATCC 90030 (without Red-EtOH), *C. albicans* ATCC 10231 and *C. krusei* ATCC 14243 with MIC = 8 mg/mL. 

It should be noted that, in addition to Red-Ac, Passion-H_2_O also showed the highest activity with a bacteriostatic effect (MBC/MIC > 4) but against strain of Gram-positive bacteria *M. luteus* ATCC 10240 (MIC = 4 mg/mL) and also very good activity with the bactericidal effect (MBC/MIC ≤ 4) against a strain of Gram-positive bacteria: *B. subtilis* ATCC 6633 (MIC = 8 mg/mL) and against a strain of Gram-negative bacteria: *B. bronchiseptica* ATCC 4617 (MIC = 8 mg/mL). 

Passion-Ac/EtOH showed almost identical activity with the fungicidal effect (MFC/MIC ≤ 4) with MIC = 8 mg/mL against the three strains of yeast: *C. albicans* ATCC 10231, *C. glabrata* ATCC 90030 and *C. krusei* ATCC 14243 as the Green-H_2_O/EtOH and differential activity against the bacterial strains. The highest activity with a bactericidal effect (MBC/MIC ≤ 4) was observed for Gram-positive bacterial strain *B. cereus* ATCC 10876 (MIC = 4 mg/mL) and a very good for Gram-negative bacterial strain *B. bronchiseptica* ATCC 4617 (MIC = 8 mg/mL) for Passion-Ac. 

Moreover, the highest activity with a bactericidal effect (MBC/MIC ≤ 4) was observed against three strains of Gram-positive bacteria: *S. aureus* ATCC 25923, *M. luteus* ATCC 10240 and *B. cereus* ATCC 10876 with MIC = 8 mg/mL and against two strains of Gram-negative bacteria: *P. aeruginosa* ATCC 27853 and *B. bronchiseptica* ATCC 4617 with MIC = 8 mg/mL for Passion-EtOH. 

Red-H_2_O showed identical activity with a bactericidal effect (MBC/MIC ≤ 4) as Green-EtOH against a strain of Gram-positive *M. luteus* ATCC 10240 (MIC = 8 mg/mL) and two strains of Gram-negative bacteria: *P. aeruginosa* ATCC 27853 (MIC = 8 mg/mL) and *B. bronchiseptica* ATCC 4617 (MIC = 4 mg/mL); however, it showed no microbial activity against yeast strains, as in the case of Green-EtOH.

The finest antimicrobial activity was characterized by acetone extracts (with the exception of Passion). The different values of the obtained results may result from the fact that medium-polar acetone is better extrahent of organic compounds present in plant material than polar water or ethanol, and thus, it can extract compounds that either increase the antibacterial activity or inhibit the action of bioactive compounds.

The extracts of edible leaves of *A. tricolor* turned out to be a much better source of bioactive compounds, showing significant antimicrobial activity (MIC = 8–16 mg/mL) compared to the results obtained by Abdoulaye et al. [41] for the methanol extract from *A. cruentus* leaves against stains of *S. aureus* ATCC 25923, *E. coli* ATCC 25922 and *P. aeruginosa* ATCC 27853 with MIC values greater than 30 mg/mL [41].

MIC for the reference antimicrobial substances were the following: MIC of vancomycin for *S. aureus* ATCC 29213 was 1 μg/mL, MIC of ciprofloxacin for *E. coli* ATCC 25922 was 0.5 μg/mL and MIC of fluconazole for *C. albicans* ATCC 10231 was 1 μg/mL. Vancomycin is a glycopeptide antibacterial developed as an alternative penicillin to treat strains of almost all Gram-positive bacteria, such as *Staphylococcus aureus* [42]. 

Ciprofloxacin is a broad spectrum fluoroquinolone antibacterial agent and is effective in the treatment of a wide variety of infections, particularly those caused by Gram-negative pathogens [43], while fluconazole is a triazole antifungal agent that is now an established part of therapy in patients with immune deficiencies [44]. 

The reference compounds show significantly higher antimicrobial activity than the tested extracts of *A*. *tricolor*, but it should be noted that the extracts have complex compositions; therefore, comparisons of pure compounds with the complex matrix should only be indicative (Table 3).

## 3. Materials and Methods

### 3.1. Reference Compounds and Reagents

Acetone (HPLC-grade), ethanol (HPLC-grade), potassium persulfate, sodium acetate trihydrate and ascorbic acid (pure p. a.) as well as formic acid (purity ≥ 98%) and hydrochloric acid (pure p.a.) were obtained from Avantor Performance Materials Poland S.A. (Gliwice, Poland).

Acetonitrile (LC-MS-grade), ABTS (2,2′-Azino-bis(3-ethylbenzthiazoline-6-sulfonic acid), Trolox (6-Hydroxy-2,5,7,8-tetramethylchroman-2-carboxylic acid), DPPH (2,2-diphenyl-1-picrylhydrazyl), TPTZ (2,4,6-tris(2-pyridyl)-s-triazine) and iron (III) chloride hexahydrate were obtained from Sigma-Aldrich (Saint Louis, MO, USA).

Reference betalains (amaranthin/isoamaranthin and betanin/isobetanin) were isolated previously from extracts of *Amaranthus cruentus* L. [11].

Reference strains of bacteria and yeast from the American Type Culture Collection (ATCC, LGC Standards, Teddington, UK) were used in the study. Gram-positive bacterial strains were *Staphylococcus aureus* ATCC 29213, *Staphylococcus aureus* ATCC 25923, *Staphylococcus aureus* ATCC 6538, *Staphylococcus aureus* ATCC BAA-1707, *Staphylococcus epidermidis* ATCC 12228, *Micrococcus luteus* ATCC 10240, *Bacillus subtilis* ATCC 6633, *Bacillus cereus* ATCC 10876 and Gram-negative bacterial strains were *Escherichia coli* ATCC 25922, *Salmonella* Typhimurium ATCC 14028, *Pseudomonas aeruginosa* ATCC 27853, *Bordetella bronchiseptica* ATCC 4617, *Klebsiella pneumoniae* ATCC 13883. The reference strains of yeast were used for *Candida albicans* ATCC 10231, *Candida glabrata* ATCC 90030, and *Candida krusei* ATCC 14243. Vancomycin, ciprofloxacin and fluconazole were obtained from Sigma-Aldrich (Saint Louis, MO, USA).

### 3.2. Plant Material and Sample Preparation

Seeds of *A. tricolor* Calaloo cv. Red, Passion and Green were purchased from Heirloom & Perennial Company in Cornwall (April 2019). Plants were planted for four weeks (May 2019). Fresh leaves (100 g) were macerated for 30 min at room temperature with water (H_2_O), ethanol (EtOH) and acetone (Ac), respectively. The extracts were filtered, partially evaporated at 25 °C under reduced pressure and freeze dried. The dried extracts were weighed and kept at −18 °C prior to phytochemical and activity experiments.

### 3.3. HPLC-DAD-ESI/HRMS Analysis

The extracts (10 mg/200 μL) were analyzed qualitatively by a high-performance-diode array detector—electrospray ionization/high-resolution mass spectrometry (HPLC-DAD-ESI/HRMS) in positive and negative ion mode. The Agilent 1260 chromatograph was equipped with an autosampler (1329B), a binary gradient pump (1312C), a column thermostat (1316A) and DAD detector (1315D). The separations were carried out on an Phenomenex Gemini RP-18 (100 mm × 2 mm; i.d. 3 μm) column in a gradient of solvents: 0.1% formic acid (solvent A) and acetonitrile with 0.1% formic acid (solvent B). The following linear gradient was adopted: 0–45 min 0–60% B; 45–46 min 60–95% B; 46–55 min 95% B, the post-time was 10 min. Total time of analysis was 65 min, with a stable flow rate at 0.200 mL/min. The injection volume was 10 μL.

A high-resolution accurate-mass quadrupole-time-of-flight mass spectrometer (HR-AM-QTOF-MS 6530B) with an ESI-Jet Stream ion source was used. The subsequent parameters of the ion source were applied: dual spray jet stream ESI (positive and negative ion mode); mass charge (*m*/*z*) range 50–1500; drying gas (N_2_) temperature 300 °C; drying gas flow 12 L/min; sheath gas temperature 325 °C; sheath gas flow 12 L/min; nebulizer pressure 35 psi, capillary voltage 4000 V; fragmentor voltage 140 V; nozzle voltage 2000 V; radiofrequency voltage 750 V; skimmer voltage 65 V; collision induced dissociation voltages 15 and 40 V. Data analysis was processed with Mass Hunter Qualitative Analysis B.08.00 software (Agilent Technologies). The assignment of the peaks noticed in the base peak chromatograms (BPC) of the nine *A. tricolor* samples was carried out by comparing the MS data with previous literature data reporting for amaranthus and other plants or online databases (PubChem, PhytoHub, MoNA, HMDB).

### 3.4. Antioxidant Activity

#### 3.4.1. ABTS Radical Scavenging Assay

The nine extracts of *A. tricolor* were assessed by the 2,2′-Azino-bis(3-ethylbenzothiazoline-6-sulfonic) acid (ABTS) radical scavenging activity. During the reaction of ABTS with sodium persulfate in the dark for 16 h, radical cation (ABTS^+•^) was obtained. 40 μL ABTS^+•^ (1 mM aqueous solution) was mixed with various volumes of aqueous extracts with a starting concentration of 10 mg/mL for the Red, Passion and Green varieties extracted with acetone (Red-Ac, Passion-Ac, Green-Ac) and for Green extracted with ethanol (Green-EtOH). The starting concentration of 5 mg/mL was for the Red variety, the extractant of which was ethanol (Red-EtOH) and for the water-extracted Passion (Passion-H_2_O), while the other extracts had starting concentration of 2.5 mg/mL. The absorbance decreases in the range of 10–90% of its initial intensity was obtained by adjusting the volume of the aqueous extracts. The final concentration of the extracts ranged from 0 to 3.5 mg/mL (for the initial concentration of 10 mg/mL), 0 to 1.3 mg/mL (for the initial concentration of 5 mg/mL) and 0 to 0.9 mg/mL (for the initial concentration of 2.5 mg/mL) in 200 μL of the total volume of each sample. In the same way, reference compounds such as Trolox (0.025 mg/mL) and ascorbic acid (0.020 mg/mL) were prepared. After 30 min of reaction kept in the dark, the absorbance of the mixture was read on a microplate reader (Infinite M200, Tecan, Austria) at λ 734 nm at 20 °C. All experiments were repeated three times. Water plus plant extracts solution was used as a blank, while 40 μL 1 mM ABTS solution plus 160 μL water was used as a negative control. The positive control was 40 μL 1 mM ABTS solution plus 0.1 mM ascorbic acid. The results were reported as mmol Trolox Equivalent per gram of dry extract (mmol TE/g DE) of each sample following the equation for Trolox y = −20.364x + 0.943 (R2 = 0.9994), which indicates how many times the given extract potential is higher or lower than the standard.

#### 3.4.2. DPPH Radical Scavenging Assay

The studied extracts were also assessed by 2,2-diphenyl-1-picrylhydrazyl (DPPH) radical scavenging assay. The references were the same as before in ABTS assay. 60 μL DPPH^•^ (1 mM ethanolic solution) was mixed with aqueous extracts with starting concentration of 30 mg/mL for Green-Ac, 20 mg/mL for Passion-H_2_O and Green-H_2_O, while the other extracts had starting concentration of 10 mg/mL. A decrease in radical absorbance in the range of 10–90% of its initial intensity was obtained by adjusting the volume of the extracts. The final concentration of the extracts ranged from 0 to 12.0 mg/mL (for the initial concentration of 30 mg/mL), 0 to 6.0 mg/mL (for the initial concentration of 20 mg/mL) and 0 to 4.0 mg/mL (for the initial concentration of 10 mg/mL) in 200 μL of the total volume of each sample. The references were prepared in the same way. After 30 min of reaction kept in the dark, spectrophotometric measurements were performed on microplate reader (Infinite M200, Tecan, Austria) at the wavelength 515 nm at 20 °C. All experiments were repeated three times. Ethanol plus plant extracts solution were used as a blank, while 60 μL 1 mM DPPH solution plus 140 μL ethanol was used as a negative control. The positive control was a 60 μL 1 mM DPPH solution plus 0.1 mM ascorbic acid. The results were reported as mmol Trolox Equivalent per gram of dry extract (mmol TE/g DE) of each sample following the equation for Trolox y = −31.873x + 1.2759 (R2 = 0.9993), which indicates how many times the given extract potential is higher or lower than the standard.

#### 3.4.3. FRAP—Ferric Reducing Antioxidant Power Assay

The ferric reducing antioxidant power method of FRAP was used to measure antioxidant activity. As the references, Trolox (0.05 mg/mL) and ascorbic acid (0.02 mg/mL) were used. The FRAP reagent was freshly prepared by mixing 300 mM buffer acetate pH 3.6 with 20 mM of FeCl_3_ × 6 H_2_O and 10 mM of TPTZ dissolved in 40 mM of hydrochloric in ratios of 10:1:1 (*v*/*v*/*v*), respectively. The 133 µL of freshly prepared FRAP reagent is added to an appropriate volume of the aqueous extract selected to ensure that the absorbance of the sample is within the range of the Trolox standard curve for a total volume of 200 µL. The starting concentration for the aqueous solution of the Green-Ac and Red-Ac varieties was 10 mg/mL, for the Green-EtOH and Red-EtOH varieties and Passion-H_2_O or Passion-Ac, the initial concentration was 5 mg/mL, while for the remaining extracts it was 2.5 mg/mL. After 10 min of reaction kept in the dark, a microplate reader (Infinite M200, Tecan, Austria) was used to spectrophotometric measurements at λ 593 nm at 20 °C. All experiments were repeated three times. Water plus the solution of plant extracts were used as a blank, while 133 µL FRAP solution plus 67 µL water was used as a negative control. The positive control was 133 µL FRAP solution plus 0.1 mM ascorbic acid. The results were reported as mmol Trolox Equivalent per gram of dry extract (mmol TE/g DE) of each sample following the equation for Trolox y = 25.739x + 0.0554 (R2 = 0.999), which indicates how many times the given extract potential is higher or lower than the standard.

### 3.5. Antimicrobial Activity

According to the recommendations of the European Committee on Antimicrobial Susceptibility Testing (EUCAST) [45], 96-well microtitrate plates were used to perform in vitro antimicrobial activity in extracts of *A. tricolor*. MIC (minimum inhibitory concentration), MBC (minimum bactericidal concentration) and MFC (minimum fungicidal concentration) were assessed. The reference strains of bacteria were subcultured on Mueller-Hinton Broth (MHB), while the yeast strains were on RPMI-1640 Medium (RPMI) and incubated for 18 to 24 h at 35 °C in ambient air. The suspension of microbial colonies was prepared according to the standard of turbidity of the bacterial suspension of 0.5 units of density according to McFarland, corresponding to 1.5 × 10^8^ CFU (colony forming units)/mL for bacteria and 5 × 10^6^ CFU/mL for yeast.

The extracts of *A. tricolor* (100 mg/mL) were dissolved in sterile distilled water. The final extracts concentrations diluted in MHB or MHB2% were ranged from 32 to 0.125 mg/mL. 2 μL of each inoculum was added to wells containing 200 μL of the serial dilution of the extracts and the plates were incubated for 18 to 24 h at 35 °C in ambient air. The Absorbance Microplate Reader EL × 800 (BioTek Instruments, Inc., Winooski, VT, USA) set at λ 580 nm was used to verify the MIC of the extracts, which showed complete inhibition of bacterial or yeast growth. The lowest extracts concentrations with no visible bacterial or yeast growth were assessed as MBC or MFC, indicating killing of 99.9% of the inoculum. MBC or MFC were evaluated by spreading 5 μL of microorganisms from each well of the microtitrate plates used for the evaluation of MIC, showing no growth onto appropriate culture medium (MHB for bacteria or RPMI for yeast). In ambient air, the plates for 18 to 24 h at 35 °C were incubated. The most frequently recurring representative value from the three measurement series was selected as the final result in determining the MIC, MBC, and MFC.

There is no statistical analysis involved in the development of the assays because they are from susceptibility tests. Vancomycin (0.06–16 μg/mL), ciprofloxacin (0.014–16 μg/mL), and fluconazole (0.06–16 μg/mL) were included as a reference antimicrobial compound.

### 3.6. Statistical Analysis

One-way analysis of variance (ANOVA) of the means of nine extracts of three *A. tricolor* Calaloo cultivars—Red, Passion and Green—was performed with Statistica, version 7.1 (StatSoft, TIBCO Software Inc. Palo Alto, CA, USA). The results were subjected to ANOVA and the differences between means were located using Fisher’s test. Significance was assessed at α level 0.05 to find out how many and which cultivars have different contents. Data were reported as the mean ± standard deviation (SD) of three measurements.

## 4. Conclusions

The high antioxidant activity was observed in the aqueous extracts, where the Red variety of *A. tricolor* showed the highest activity in all the assays. A strong bacteriostatic, bactericidal and fungicidal effect against selected Gram-positive bacteria strains *S. aureus*, *B. subtilis*, *M. luteus* and *B. cereus* causing food poisoning in humans, as well as Gram-negative bacterium *B. bronchiseptica* responsible for mild forms of respiratory diseases in humans and yeast strain *C. krusei* was noticeable in the Red acetone extract of *A. tricolor*. Selected metabolites, such as *N*-(carboxyacetyl) phenylalanine, dopa, norleucine, tryptophan, quercetin-*O*-hexoside, luteolin-*O*-rhamnosylhexoside, luteolin-6-*C*-hexoside, succinic acid, gallic acid-*O*-hexoside, dihydroxybenzoic acid and hydroxybenzoic acid show antioxidant and antibacterial activities. The highest content of these compounds was identified in the Green variety of *A. tricolor* extracted with acetone. The effectiveness of these compounds may be higher against microbes, as the presence of antioxidants may reduce the inflammatory processes that accompany microbial diseases. The greatest effect on the antifungal activity against fungal exhibited maleic acid, for which no antioxidant activity was noticed. The Passion/Green-EtOH were the richest sources of maleic acid.

Research on the activity of pure compounds requires a considerable amount of time to separate compounds from a complex plant matrix. This study provides a tool for fast screening of plant material in search of biologically active compounds. Furthermore, this study indicates the richest source of active compounds. The activity of the compounds may not be related to their content in the extract, because the matrix components can negatively or positively affect their activities. Therefore, the measure of extract activity will not indicate the potential of the plant.

## Figures and Tables

**Table 1:** High-resolution mass spectrometric data obtained by HPLC-DAD-ESI-HRMS/MS for metabolites of three cultivars of *A. tricolor* Calaloo (Red, Passion and Green) analyzed in negative and positive ionization modes.

No.	Metabolite	Molecular Formula	t_R_ (min)	*m*/*z* (M − H)^−^_exp_	*m*/*z* (M + H)^+^_exp_	*m*/*z* (M ± H)^±^ _calc_	Δ (ppm)	*m*/*z* from MS^2^ of (M − H)^−^	Ref.
	**Amino acids**								
1	Serine	C_3_H_7_NO_3_	1.6	104.0356		104.0353	−2.70	60.0441	[13]
2	Aspartic acid	C_4_H_7_NO_4_	1.7	132.0301		132.0302	0.99	88.0422	[14]
3	Tyrosine	C_9_H_11_NO_3_	12.9	180.0662		180.0666	2.30	119.0348	[14]
4	*N*-benzoylaspartic acid	C_11_H_11_NO_5_	13.1	236.0564		236.0564	0.19	192.0695; 174.9560	[15]
5	Glutamic acid	C_5_H_9_NO_4_	13.4	146.0460		146.0459	−0.81	102.0559	[14]
6	Isoleucine	C_6_H_13_NO_2_	13.7	130.0875		130.0874	−1.13	69.0693	[14]
7	*N*-(carboxyacetyl) phenylalanine	C_12_H_13_NO_5_	14.2	250.0720		250.0721	0.38	206.0824	[15]
8	Dopa	C_9_H_11_NO_4_	14.7	196.0624		196.0615	−4.41	179.0022; 135.0557	[16]
9	Leucine	C_6_H_13_NO_2_	18.1	130.0880		130.0874	−4.94	69.0686	[14]
10	Phenylalanine	C_9_H_11_NO_2_	18.3	164.0710		164.0717	4.25	147.0432	[14]
11	Tryptophan	C_11_H_12_N_2_O_2_	22.3	203.0826		203.0826	0.01	142.0651	[17]
12	Norleucine	C_6_H_13_NO_2_	26.2	130.0873		130.0874	0.40	69.0689	[14]
	**Betacyanins**								
13	Betanidin-5-*O*-*ß*-glucuronosylglucoside (Amaranthin)	C_30_H_34_N_2_O_19_	8.5		727.1826	727.1829	0.35	551.1512; 389.1637	[28]
14	Betanidin-5-*O*-*ß*-glucoside (Betanin)	C_24_H_26_N_2_O_13_	9.9		551.1508	551.1508	−0.06	389.1640	[18]
15	Isobetanidin-5-*O*-*ß*-glucuronosylglucoside (Isoamaranthin)	C_30_H_34_N_2_O_19_	10.6		727.1832	727.1829	−0.48	551.1507; 389.1632	[28]
16	Isobetanidin-5-*O*-*ß*-glucoside (Isobetanin)	C_24_H_26_N_2_O_13_	10.8		551.1516	551.1508	−1.52	389.1634	[18]
	**Fatty acids**								
17	Tuberonic acid hexoside	C_18_H_28_O_9_	13.7	387.1673		387.1661	−3.20	225.1132; 207.0976; 163.0400	[19]
18	Tuberonic acid	C_12_H_18_O_4_	16.0	225.1127		225.1132	2.36	163.0425;	[19]
19	Trihydroxyoctadecadienoic acid	C_18_H_32_O_5_	32.0	327.2181		327.2177	−1.23	299.0780; 271.1568; 229.1437; 171.1008	[19]
20	Hydroperoxyoctadecadienoic acid	C_18_H_32_O_4_	44.5	311.2233		311.2228	−1.66	293.1811; 161.0768	[19]
21	Hydroxyoctadecatrienoic acid I	C_18_H_30_O_3_	46.0	293.2129		293.2122	−2.32	275.2002; 183.1435; 171.0994; 121.1026	[19]
22	Hydroxyoctadecatrienoic acid II	C_18_H_30_O_3_	46.8	293.2123		293.2122	−0.28	275.2002; 183.1435; 171.0992; 121.1021	[19]
23	Hydroxyoctadecatrienoic acid III	C_18_H_30_O_3_	47.1	293.2128		293.2122	−1.98	275.1915; 183.1427; 171.0987; 121.1018	[19]
24	Hydroxyoctadecadienoic acid I	C_18_H_32_O_3_	49.4	295.2279		295.2279	−0.11	277.2162; 181.0419; 171.9492	[19]
25	Hydroxyoctadecadienoic acid II	C_18_H_32_O_3_	52.0	295.2280		295.2279	−0.44	277.2162; 181.0410; 171.9496	[19]
	**Flavonoids**								
26	Quercetin-*O*-hexoside	C_21_H_20_O_12_	23.7	463.0886		463.0882	−0.86	301.0333	[19]
27	Luteolin-*O*-rhamnosylhexoside	C_27_H_30_O_15_	24.7	593.1514		593.1512	−0.35	447.0893; 285.0180	[19]
28	Luteolin-6-*C*-hexoside (homoorientin)	C_21_H_20_O_11_	25.4	447.0944		447.0933	−2.49	429.1788; 357.1467; 327.1175; 297.1072; 285.1535; 213.0244; 133.0106	[21]
29	Phlorizin	C_21_H_24_O_10_	28.8	435.1301		435.1297	−0.98	329.1624; 273.1210; 167.0340	[21]
30	Luteolin-7-*O*-glucoside	C_21_H_20_O_11_	41.4	447.0933		447.0933	−0.03	285.0174; 243.0144; 199.8430; 175.9898	[21]
	**Organic acids**								
31	Citric acid	C_6_H_8_O_7_	1.9	191.0199		191.0197	−0.91	111.0072	[22,23]
32	Mannonic acid	C_6_H_12_O_7_	1.9	195.0510		195.0510	0.13	159.0855	[9]
33	Malic acid	C_4_H_6_O_5_	2.1	133.0134		133.0142	6.32	115.0027	[15]
34	Maleic acid	C_4_H_4_O_4_	2.1	115.0037		115.0037	−0.15	71.0156	[9]
35	Succinic acid	C_4_H_6_O_4_	2.5	117.0193		117.0193	0.27	73.0036	[9,24]
36	Glyceric acid	C_3_H_6_O_4_	2.9	105.0192		105.0193	1.25	73.0005	[9]
37	2-Oxopentanoic acid	C_5_H_8_O_3_	9.2	115.0402		115.0401	−1.14	71.1022	[9]
38	Fumaric acid	C_4_H_4_O_4_	23.4	115.0037		115.0037	−0.15	71.1020	[9,24]
	**Phenolic acids**								
39	Gallic acid	C_7_H_6_O_5_	3.7	169.0152		169.0142	−5.61	125.0247	[25]
40	Gallic acid-*O*-hexoside	C_13_H_16_O_16_	5.7	331.0667		331.0671	1.12	237.0881; 169.0247; 151.0382; 125.0247; 123.0442; 115.0385	[21]
41	Vanillic acid-*O*-hexoside	C_14_H_18_O_9_	6.1	329.0878		329.0879	−0.29	167.0345; 123.0442	[19]
42	Vanillic acid	C_8_H_8_O_4_	6.4	167.0345		167.0350	2.87	152.9122; 123.0442; 108.0206	[21]
43	Protocatechuic acid-*O*-hexoside	C_13_H_16_O_9_	7.4	315.0729		315.0722	−2.35	153.0197; 109.0304	[21]
44	Dihydroxybenzoic acid	C_7_H_6_O_4_	7.4	153.0195		153.0193	−1.09	121.0298; 77.1045	[19]
45	Hydroxybenzoic acid	C_7_H_6_O_3_	9.9	137.0244		137.0244	0.13	121.0298; 77.1045	[19]
46	Benzoic acid	C_7_H_6_O_2_	13.5	121.0298		121.0295	−2.43	77.1045	[19]
47	Ferulic acid	C_10_H_10_O_4_	13.5	193.0503		193.0506	1.71	178.0280; 149.0626	[15]
48	*p*-Coumaric acid	C_9_H_8_O_3_	13.8	163.0401		163.0401	−0.20	119.0500	[15]
49	*o*-Coumaric acid	C_9_H_8_O_3_	14.2	163.0401		163.0401	−0.20	135.0453; 119.0502	[21]
50	Syringic acid	C_9_H_10_O_5_	17.1	197.0457		197.0455	−0.77	153.1034; 179.0488; 135.0312	[17]
51	Cinnamic acid	C_9_H_8_O_2_	18.4	147.0452		147.0452	−0.32	103.0530	[26]
52	Caffeic acid	C_9_H_8_O_4_	19.8	179.0350		179.0350	−0.10	135.0430	[21]

**Table 2:** Antioxidant properties of three cultivars of *A. tricolor* Calaloo (Red, Passion and Green) assessed by the ABTS, FRAP and DPPH assays, and correlation coefficients between identified metabolites (absolute peak areas) and antioxidant activity (mmol TE/g DE). Statistical significance is marked by font: boldface means 95% significance and very strong correlation (R = 0.7–1.0), and italic font means 95% significance and strong correlation (R = 0.5–0.7).

Antioxidant Activity			ABTS		FRAP		DPPH	
			[mmol TE/g DE] *					
**Red**		H_2_O	0.060 ^h^	±0.012	0.053 ^h^	±0.028	0.021 ^h^	±0.027
		EtOH	0.035 ^d^	±0.011	0.030 ^f^	±0.033	0.019 ^f^	±0.024
		Ac	0.020 ^c^	±0.008	0.017 ^d^	±0.010	0.017 ^e^	±0.011
**Passion**		H_2_O	0.056 ^f^	±0.018	0.023 ^e^	±0.011	0.016 ^d^	±0.024
		EtOH	0.048 ^e^	±0.006	0.015 ^c^	±0.009	0.014 ^c^	±0.012
		Ac	0.018 ^b^	±0.010	0.013 ^b^	±0.029	0.011 ^b^	±0.015
**Green**		H_2_O	0.058 ^g^	±0.012	0.035 ^g^	±0.014	0.020 ^g^	±0.049
		EtOH	0.018 ^b^	±0.010	0.015 ^c^	±0.008	0.014 ^c^	±0.059
		Ac	0.015 ^a^	±0.015	0.009 ^a^	±0.019	0.009 ^a^	±0.051
**Ascorbic acid**			7.8	±0.035	4.6	±0.010	8.1	±0.040
**Fisher’ LSD**			0.003		0.002		0.002	
	**Correlation**							
***No.***	***Amino acids***							
1	Serine		−0.373		−0.412		−0.430	
2	Aspartic acid		−0.452		−0.500		−0.509	
3	Tyrosine		−0.050		−0.156		0.001	
4	*N*-benzoylaspartic acid		0.095		−0.160		−0.223	
5	Glutamic acid		0.200		*0.539*		*0.524*	
6	Isoleucine		0.314		0.234		0.035	
7	*N*-(carboxyacetyl) phenylalanine		0.161		0.407		0.285	
8	Dopa		*0.550*		*0.690*		*0.640*	
9	Leucine		0.109		0.0004		−0.122	
10	Phenylalanine		0.012		−0.314		−0.302	
11	Tryptophan		0.005		0.353		0.179	
12	Norleucine		0.322		*0.595*		0.430	
	***Betacyanins***							
13	Betanidin-5-*O*-*ß*-glucuronosylglucoside (Amaranthin)		−0.019		−0.382		−0.353	
14	Betanidin-5-*O*-*ß*-glucoside (Betanin)		−0.184		−0.471		−0.539	
15	Isobetanidin-5-*O*-*ß*-glucuronosylglucoside (Isoamaranthin)		−0.529		−0.585		−0.671	
16	Isobetanidin-5-*O*-*ß*-glucoside (Isobetanin)		−0.425		−0.487		−0.583	
	***Fatty acids***							
17	Tuberonic acid hexoside		0.487		**0.755**		*0.678*	
18	Tuberonic acid		−0.183		−0.047		−0.103	
19	Trihydroxyoctadecadienoic acid		−0.267		0.068		0.239	
20	Hydroperoxyoctadecadienoic acid		−0.172		−0.432		−0.296	
21	Hydroxyoctadecatrienoic acid I		−0.092		−0.202		−0.037	
22	Hydroxyoctadecatrienoic acid II		−0.217		−0.118		0.103	
23	Hydroxyoctadecatrienoic acid III		−0.162		−0.213		0.008	
24	Hydroxyoctadecadienoic acid I		−0.440		−0.416		−0.152	
25	Hydroxyoctadecadienoic acid II		−0.633		−0.663		−0.681	
	***Flavonoids***							
26	Quercetin-*O*-hexoside		*0.590*		**0.775**		*0.500*	
27	Luteolin-*O*-rhamnosylhexoside		0.464		0.474		0.277	
28	Luteolin-6-*C*-hexoside (homoorientin)		**0.765**		**0.885**		**0.703**	
29	Phlorizin		0.304		0.392		0.364	
30	Luteolin-7-*O*-glucoside		0.180		−0.086		−0.140	
	***Organic*** ***acids***							
31	Citric acid		−0.199		−0.300		−0.223	
32	Mannonic acid		−0.394		−0.593		−0.625	
33	Malic acid		−0.722		−0.669		−0.509	
34	Maleic acid		−0.704		−0.497		−0.489	
35	Succinic acid		0.241		0.374		0.351	
36	Glyceric acid		−0.408		−0.340		−0.245	
37	2-Oxopentanoic acid		0.124		−0.083		−0.102	
38	Fumaric acid		−0.296		−0.031		0.056	
	***Phenolic*** ***acids***							
39	Gallic acid		−0.020		−0.037		−0.167	
40	Gallic acid-*O*-hexoside		*0.629*		*0.649*		*0.541*	
41	Vanillic acid-*O*-hexoside		0.069		−0.140		−0.041	
42	Vanillic acid		*0.549*		0.412		0.332	
43	Protocatechuic acid-*O*-hexoside		0.138		0.056		0.002	
44	Dihydroxybenzoic acid		*0.535*		0.427		0.339	
45	Hydroxybenzoic acid		**0.708**		**0.772**		*0.546*	
46	Benzoic acid		0.261		−0.100		−0.192	
47	Ferulic acid		−0.107		−0.085		−0.061	
48	*p*-Coumaric acid		0.196		*0.502*		0.465	
49	*o*-Coumaric acid		0.167		0.439		0.427	
50	Syringic acid		0.209		0.156		0.115	
51	Cinnamic acid		−0.024		−0.083		−0.105	
52	Caffeic acid		0.362		0.423		0.299	

* DE = Dry Extract Weight; TE = Trolox Equivalent. Each value represents the mean and ± standard deviation from three lots. Values in each row with the same letter are not significantly different at *p* ˂ 0.05. The correlation is significant at the 0.05 level.

**Table 3:** Antimicrobial activity of three cultivars of *A. tricolor* Calaloo (Red, Passion and Green) assessed as the MIC (minimum inhibitory concentration), MBC (minimum bactericidal concentration), MFC (minimum fungicidal concentration) and correlation coefficients between identified metabolites (absolute peak areas) and microbial activity (MIC values).

		Gram-Positive Bacteria								Gram-Negative Bacteria					Fungal Strains		
Microrganism/ Metabolite		*S.* *aureus* ATCC 29213	*S. aureus* ATCC 25923	*S. aureus* ATCC 6538	*S. aureus* ATCC BAA-1707	*S. epidermidis* ATCC 12228	*M.* *luteus* ATCC 10240	*B. subtilis* ATCC 6633	*B. cereus* ATCC 10876	*E.* *coli* ATCC 25922	*S. Typhimurium* ATCC 14028	*P. aeruginosa* ATCC 27853	*B. bronchiseptica* ATCC 4617	*K. pneumoniae* ATCC 13883	*C. albicans* ATCC 10231	*C. glabrata* ATCC 90030	*C. krusei* ATCC 14243
		MIC; MBC (MCB/MIC)								MIC; MBC (MCB/MIC)					MIC; MFC (MFC/MIC)		
**Red**	H_2_O	32; 32 (1)	16; 32 (2)	32; 32 (1)	32; 32 (1)	16; 32 (2)	8; 32 (4)	16; 16 (1)	16; 16 (1)	16; 16 (1)	16; 32 (2)	8; 16 (2)	4; 16 (4)	16; 32 (2)	16; 16 (1)	16; 32 (2)	16; 6 (1)
	EtOH	16; 16 (1)	32; 32 (1)	16; 32 (2)	32; 32 (1)	32; 32 (1)	8; 32 (4)	16; 16 (1)	16; 16 (1)	16; 16 (1)	16; 32 (2)	8; 16 (2)	4; 8 (2)	16; 32 (2)	8; 8 (1)	16; 16 (1)	8; 8 (1)
	Ac	4; 32 (8)	8; 16 (2)	4; 32 (8)	32; 32 (1)	32; 32 (1)	8; 16 (2)	8; 16 (2)	4; 16 (4)	16; 16 (1)	32; 32 (1)	16; 16 (1)	4; 8 (2)	16; 32 (2)	16; 16 (1)	16; 16 (1)	8; 8 (1)
**Passion**	H_2_O	16; 32 (2)	16; 32 (2)	16; 32 (2)	16; 32 (2)	16; 32 (2)	4; 32 (8)	8; 32 (4)	16; 32 (2)	16; 32 (2)	16; 32 (2)	16; 32 (2)	8; 32 (4)	16; 32 (2)	16; 16 (1)	16; 32 (2)	16; 32 (2)
	EtOH	32; 32 (1)	8; 32 (4)	32; 32 (1)	32; 32 (1)	32; 32 (1)	8; 32 (4)	16; 16 (1)	8; 16 (2)	16; 16 (1)	32; 32 (1)	8; 16 (2)	8; 8 (1)	32; 32 (1)	8; 8 (1)	8; 16 (2)	8; 8 (1)
	Ac	16; 16 (1)	16; 32 (2)	32; 32 (1)	16; 16 (1)	32; 32 (1)	32; 32 (1)	16; 16 (1)	4; 16 (4)	16; 16 (1)	32; 32 (1)	16; 16 (1)	8; 16 (2)	32; 32 (1)	8; 8 (1)	8; 16 (2)	8; 16 (2)
**Green**	H_2_O	32; 32 (1)	32; 32 (1)	32; 32 (1)	16; 32 (2)	16; 32 (2)	8; 32 (4)	16; 16 (1)	16; 16 (1)	16; 16 (1)	16; 32 (2)	8; 16 (2)	4; 16 (4)	32; 32 (1)	8; 16 (2)	8; 16 (2)	8; 16 (2)
	EtOH	16; 16 (1)	16; 16 (1)	16; 32 (2)	16; 16 (1)	16; 16 (1)	8; 32 (4)	16; 16 (1)	16; 16 (1)	16; 16 (1)	16; 32 (2)	8; 16 (2)	4; 16 (4)	16; 32 (2)	8; 8 (1)	8; 16 (2)	8; 16 (2)
	Ac	16; 16 (1)	8; 32 (4)	8; 32 (4)	8; 16 (2)	8; 32 (4)	32; 32 (1)	8; 16 (2)	8; 16 (2)	16; 16 (1)	16; 32 (2)	8; 16 (2)	16; 16 (1)	16; 32 (2)	8; 8 (1)	8; 16 (2)	16; 16 (1)
**Correlation**																	
**Amino acids**																	
Serine		0.096	0.367	0.348	0.595	0.524	**−0.449**	**−0.117**	**−0.167**	0.199	0.473	0.199	0.387	0.061	0.472	**−0.383**	**−0.270**
Aspartic acid		0.047	0.461	0.328	0.547	0.502	**−0.401**	**−0.138**	**−0.160**	0.263	0.456	0.263	0.260	0.031	0.454	**−0.510**	**−0.404**
Tyrosine		0.317	0.350	**−0.017**	**−0.302**	0.440	0.291	0.280	**−0.069**	0.359	**−0.053**	0.359	**−0.184**	**−0.201**	**−0.053**	**−0.376**	0.093
N-benzoylaspartic acid		**−0.431**	0.068	0.082	**−0.304**	**−0.319**	0.199	**−0.141**	0.281	0.037	**−0.055**	0.037	**−0.285**	0.071	0.016	**−0.072**	**−0.389**
Glutamic acid		**−0.252**	**−0.151**	**−0.109**	**−0.250**	0.065	**−0.107**	0.576	**−0.060**	**−0.716**	0.127	**−0.716**	0.127	**−0.375**	**−0.387**	0.741	0.603
Isoleucine		**−0.331**	**−0.331**	0.180	0.142	**−0.547**	**−0.125**	**−0.296**	0.326	**−0.376**	**−0.030**	**−0.376**	0.090	0.271	0.150	0.391	**−0.133**
N-(carboxyacetyl) phenylalanine		**−0.424**	**−0.524**	**−0.295**	**−0.071**	**−0.259**	**−0.110**	0.242	**−0.154**	**−0.650**	**−0.047**	**−0.650**	0.237	0.027	**−0.544**	0.839	0.601
Dopa		0.425	**−0.125**	0.074	**−0.235**	**−0.442**	0.372	**−0.015**	0.324	**−0.371**	**−0.462**	**−0.371**	**−0.193**	**−0.185**	**−0.065**	0.320	0.264
Leucine		0.185	**−0.332**	**−0.075**	0.272	**−0.684**	0.144	**−0.675**	0.206	0.211	**−0.456**	0.211	**−0.231**	0.502	0.127	**−0.220**	**−0.361**
Phenylalanine		**−0.555**	**−0.384**	**−0.070**	**−0.211**	**−0.139**	**−0.078**	**−0.227**	**−0.122**	0.165	0.200	0.165	0.490	0.143	**−0.015**	0.385	**−0.036**
Tryptophan		**−0.296**	**−0.368**	**−0.321**	0.180	**−0.358**	**−0.128**	0.141	**−0.061**	**−0.587**	**−0.178**	**−0.587**	**−0.178**	0.194	**−0.495**	0.422	0.321
Norleucine		**−0.082**	**−0.159**	0.012	0.002	**−0.399**	0.030	0.144	0.242	**−0.685**	**−0.202**	**−0.685**	**−0.202**	**−0.047**	**−0.214**	0.450	0.242
**Betacyanins**																	
Betanidin-5-O-ß-glucuronosylglucoside (Amaranthin)		**−0.404**	0.331	0.439	0.055	0.429	**−0.377**	0.221	0.377	0.203	0.608	0.203	**−0.132**	0.190	0.611	**−0.383**	**−0.491**
Betanidin-5-O-ß-glucoside (Betanin)		**−0.285**	0.304	0.499	0.516	0.509	**−0.570**	0.020	0.171	0.162	0.659	0.162	0.201	0.344	0.654	**−0.387**	**−0.423**
Isobetanidin-5-O-ß-glucuronosylglucoside (Isoamaranthin)		**−0.179**	0.272	0.138	0.491	0.054	**−0.249**	**−0.476**	**−0.233**	0.259	0.223	0.259	0.223	0.118	0.223	**−0.414**	**−0.557**
Isobetanidin-5-O-ß-glucoside (Isobetanin)		**−0.103**	0.202	0.318	0.752	0.307	**−0.558**	**−0.329**	**−0.170**	0.157	0.477	0.157	0.450	0.249	0.477	**−0.314**	**−0.397**
**Fatty acids**																	
Tuberonic acid hexoside		0.483	0.029	0.049	**−0.175**	**−0.324**	0.382	0.178	0.316	**−0.493**	**−0.494**	**−0.493**	**−0.392**	**−0.213**	**−0.185**	0.211	0.341
Tuberonic acid		**−0.155**	**−0.660**	**−0.466**	0.347	**−0.504**	**−0.221**	**−0.398**	**−0.200**	0.066	**−0.202**	0.066	0.009	0.493	**−0.210**	0.136	**−0.027**
Trihydroxyoctadecadienoic acid		0.011	**−0.487**	**−0.606**	0.034	0.004	**−0.249**	0.163	**−0.401**	0.079	**−0.006**	0.079	0.004	0.025	**−0.340**	0.195	0.308
Hydroperoxyoctadecadienoic acid		**−0.381**	0.196	0.039	**−0.249**	0.259	**−0.115**	0.164	0.112	0.372	0.347	0.372	**−0.228**	0.016	0.220	**−0.343**	**−0.407**
Hydroxyoctadecatrienoic acid I		0.115	0.363	**−0.050**	**−0.357**	0.336	0.227	0.308	0.061	0.364	0.001	0.364	**−0.444**	**−0.155**	**−0.008**	**−0.477**	**−0.131**
Hydroxyoctadecatrienoic acid II		0.302	0.202	**−0.319**	**−0.270**	0.377	0.188	0.372	**−0.179**	0.376	**−0.101**	0.376	**−0.417**	**−0.170**	**−0.221**	**−0.424**	0.110
Hydroxyoctadecatrienoic acid III		0.176	0.118	**−0.300**	**−0.349**	0.321	0.211	0.294	**−0.132**	0.469	**−0.078**	0.469	**−0.359**	**−0.077**	**−0.175**	**−0.391**	0.050
Hydroxyoctadecadienoic acid I		0.062	0.430	**−0.045**	**−0.161**	0.643	**−0.127**	0.284	**−0.291**	0.442	0.384	0.442	0.019	**−0.404**	0.125	**−0.411**	**−0.171**
Hydroxyoctadecatdienoic acid II		**−0.078**	0.352	**−0.017**	0.462	0.079	**−0.199**	**−0.376**	**−0.145**	0.480	0.119	0.480	**−0.272**	0.242	0.210	**−0.818**	**−0.729**
**Flavonoids**																	
Quercetin-O-hexoside		**−0.053**	0.190	0.346	**−0.179**	**−0.243**	0.289	0.372	0.421	**−0.996**	**−0.235**	**−0.996**	**−0.172**	**−0.253**	**−0.264**	0.568	0.439
Luteolin-O-rhamnosylhexoside		**−0.060**	**−0.071**	0.336	**−0.033**	**−0.116**	0.082	0.074	0.135	**−0.660**	**−0.018**	**−0.660**	0.476	**−0.160**	**−0.071**	0.706	0.461
Luteolin-6-C-hexoside (homoorientin)		0.306	0.265	0.482	**−0.306**	**−0.359**	0.440	0.200	0.615	**−0.788**	**−0.354**	**−0.788**	**−0.268**	**−0.392**	0.037	0.403	0.242
Phlorizin		**−0.311**	0.048	0.249	**−0.326**	0.023	**−0.059**	0.324	0.123	**−0.596**	0.262	−0.596	0.316	**−0.483**	**−0.015**	0.678	0.266
Luteolin-7-O-glucoside		0.106	**−0.090**	0.344	0.167	0.050	**−0.062**	**−0.342**	**−0.019**	0.088	0.138	0.088	0.694	0.064	0.343	0.222	0.046
**Organic acids**																	
Citric acid		**−0.121**	0.420	0.022	**−0.206**	0.185	0.109	0.164	0.125	0.270	0.073	0.270	**−0.509**	**−0.077**	0.058	**−0.534**	**−0.396**
Mannonic acid		**−0.169**	0.351	0.383	0.487	0.342	**−0.422**	**−0.303**	**−0.078**	0.309	0.509	0.309	0.351	0.116	0.552	**−0.424**	**−0.559**
Malic acid		0.142	0.042	**−0.297**	0.486	0.473	**−0.384**	**−0.142**	**−0.524**	0.677	0.255	0.677	0.118	0.236	0.094	**−0.620**	**−0.231**
Maleic acid		**−0.130**	**−0.437**	**−0.615**	0.660	0.017	**−0.455**	**−0.258**	**−0.575**	0.372	0.026	0.372	0.030	0.590	**−0.277**	**−0.299**	**−0.058**
Succinic acid		**−0.170**	**−0.622**	**−0.255**	**−0.130**	**−0.321**	**−0.047**	**−0.007**	**−0.161**	**−0.364**	**−0.093**	**−0.364**	0.433	**−0.023**	**−0.319**	0.811	0.517
Glyceric acid		**−0.274**	**−0.366**	**−0.127**	0.397	0.301	**−0.661**	**−0.142**	**−0.536**	0.135	0.567	0.135	0.879	0.032	0.150	0.346	0.100
2-Oxopentanoic acid		**−0.004**	0.148	0.562	0.232	0.190	**−0.320**	**−0.211**	0.149	**−0.002**	0.451	**−0.002**	0.588	**−0.095**	0.659	0.106	**−0.231**
Fumaric acid		0.033	0.195	0.115	0.302	0.624	**−0.505**	0.334	**−0.338**	**−0.168**	0.547	**−0.168**	0.494	**−0.331**	0.145	0.145	0.222
**Phenolic acids**																	
Gallic acid		0.150	**−0.223**	**−0.071**	0.399	**−0.615**	0.021	**−0.609**	0.208	0.173	**−0.374**	0.173	**−0.365**	0.507	0.159	**−0.357**	**−0.466**
Gallic acid-O-hexoside		0.122	0.003	0.363	**−0.249**	**−0.413**	0.227	0.004	0.505	**−0.532**	**−0.191**	**−0.532**	**−0.071**	**−0.243**	0.167	0.431	0.081
Vanillic acid-O-hexoside		0.119	**−0.378**	0.055	0.076	**−0.081**	**−0.151**	**−0.444**	**−0.139**	0.344	0.128	0.344	0.665	0.067	0.347	0.228	**−0.036**
Vanillic acid		0.078	**−0.143**	0.411	**−0.087**	**−0.179**	0.027	**−0.096**	0.304	**−0.340**	0.041	**−0.340**	0.432	**−0.111**	0.303	0.532	0.185
Protocatechuic acid-O-hexoside		**−0.192**	**−0.329**	0.097	0.065	0.080	**−0.186**	**−0.060**	**−0.225**	**−0.222**	0.229	**−0.222**	0.837	**−0.026**	**−0.022**	0.668	0.418
Dihydroxybenzoic acid		0.225	**−0.225**	0.268	**−0.119**	**−0.361**	0.221	**−0.268**	0.213	**−0.259**	**−0.214**	**−0.259**	0.398	**−0.094**	0.141	0.509	0.223
Hydroxybenzoic acid		0.108	0.012	0.389	**−0.202**	**−0.364**	0.323	0.109	0.420	**−0.806**	**−0.279**	**−0.806**	0.086	**−0.236**	**−0.082**	0.654	0.410
Benzoic acid		**−0.249**	0.272	0.581	**−0.092**	**−0.129**	0.012	**−0.249**	0.442	**−0.031**	0.223	**−0.031**	0.116	**−0.051**	0.538	**−0.028**	**−0.492**
Ferulic acid		**−0.356**	**−0.260**	0.052	0.131	0.243	**−0.454**	0.034	**−0.337**	**−0.184**	0.506	**−0.184**	0.859	**−0.145**	0.066	0.607	0.267
p-Coumaric acid		**−0.137**	**−0.098**	**−0.014**	**−0.141**	0.217	**−0.114**	0.553	**−0.154**	**−0.708**	0.164	**−0.708**	0.366	**−0.382**	**−0.357**	0.769	0.727
o-Coumaric acid		**−0.208**	**−0.185**	**−0.047**	**−0.149**	0.213	**−0.175**	0.508	**−0.201**	**−0.648**	0.227	**−0.648**	0.469	**−0.363**	**−0.331**	0.824	0.710
Syringic acid		0.429	0.037	0.190	0.147	**−0.466**	0.173	**−0.479**	0.378	0.162	**−0.335**	0.162	**−0.334**	0.140	0.388	**−0.350**	**−0.448**
Cinnamic acid		**−0.660**	**−0.206**	**−0.004**	0.104	0.104	**−0.532**	0.340	0.224	**−0.173**	0.476	**−0.173**	**−0.185**	0.342	0.157	0.098	**−0.134**
Caffeic acid		**−0.021**	**−0.077**	0.269	**−0.030**	0.061	**−0.010**	0.168	**−0.006**	**−0.585**	0.092	**−0.585**	0.586	**−0.242**	**−0.077**	0.715	0.543

MIC, MBC and MFC were expressed as mg/mL. The representative data (mode) are presented.

## Data Availability

Not applicable.

## References

[1] Miguel M.G. (2018). Betalains in some species of the Amaranthaceae family: A Review. Antioxidants.

[2] Haber T., Obiedziński M., Waszkiewicz-Robak B., Biller E., Achremowicz B., Ceglińska A. (2017). Pseudocereals and the possibilities of their application in food technology *Amaranth* and *Quinoa* application in food processing. Pol. J. Appl. Sci..

[3] Rahman A.H.M.M., Gulshana M.I.A. (2014). Taxonomy and medicinal uses on Amaranthaceae family of Rajshahi, Bangladesh. Appl. Ecol. Environ. Sci..

[4] Nahar K., Kabir F., Islam P., Rahman M.M., Al Mamun M.A., Faruk M., Subhan N., Rahman G.M.S., Reza H.M., Alam M.A. (2018). Cardioprotective effect of *Amaranthus tricolor* extract in isoprenaline induced myocardial damage in ovariectomized rats. Biomed. Pharmacother..

[5] Samsul A., Krupanidhi K., Sambasiva Rao K.R.S. (2013). Evaluation of *in-vitro* antioxidant activity of *Amaranthus tricolor* Linn. Asian J. Pharmacol. Toxicol..

[6] Angerhofer C.K., Maes D., Giacomoni P.U. (2009). The Use of Natural Compounds and Botanicals in the Development of Anti-Aging Skin Care Products. Skin Aging Handbook.

[7] Hunyadi A. (2019). Themechanism(s) of action of antioxidants:from scavenging reactive oxygen/nitrogen species to redox signaling and the generation of bioactive secondary metabolites. Med. Res. Rev..

[8] Motyleva S., Gins M., Gins V., Tetyannikov N., Kulikov I., Kabashnikova L., Panischeva D., Mertvischeva M., Domanskaya I. (2022). Metabolite profile of Amaranthus tricolor *L.* and Amaranthus cruentus *L.* in Adaptation to Drought.

[9] Castrillón-Arbeláez P.A., Frier J.P.D. (2016). Secondary Metabolism in Amaranthus spp.-A Genomic Approach to Understand its Diversity and Responsiveness to Stress in Marginally Studied Crops with Highagronomic Potential.

[10] Cai Y., Sun M., Corke H. (2003). Antioxidant activity of betalains from plants of the Amaranthaceae. J. Agric. Food Chem..

[11] Spórna-Kucab A., Kumorkiewicz A., Szmyr N., Szneler E., Wybraniec S. (2019). Separation of betacyanins from flowers of *Amaranthus cruentus* L. in a polar solvent system by high-speed counter-current chromatography. J. Sep. Sci..

[12] Jeong W.T., Bang J.H., Han S., Hyun T.K., Cho H., Lim H.B., Chung J.W. (2020). Establishment of a UPLC-PDA/ESI-Q-TOF/MS-based approach for the simultaneous analysis of multiple phenolic compounds in Amaranth (*A. cruentus* and *A. tricolor*). Molecules.

[13] Sugimoto H., Kakehi M., Jinno F. (2015). Bioanalytical Method for the Simultaneous Determination of *D*- and *L*-serine in human plasma by LC/MS/MS. Anal. Biochem..

[14] Harder U., Koletzko B., Peissner W. (2011). Quantification of 22 plasma amino acids combining derivatization and ion-pair LC–MS/MS. J. Chromatogr. B Biomed. Appl..

[15] Zhang H., Chen G., Yang J., Yang C., Guo M. (2021). Screening and characterisation of potential antioxidant, hypoglycemic and hypolipidemic components revealed in *Portulaca oleracea* via multi-target affinity ultrafiltration LC–MS and molecular docking. Phytochem Anal..

[16] Xing J., Yang Z., Lv B., Xiang L. (2008). Rapid screening for cyclo-dopa and diketopiperazine alkaloids in crude extracts of *Portulaca oleracea* L. using liquid chromatography/tandem mass spectrometry. Rapid Commun. Mass Spectrom..

[17] Taamalli A., Arráez-Román D., Abaza L., Iswaldi I., Fernández-Gutiérrez A., Zarrouk M., Segura-Carretero A. (2015). LC-MS-basedmetabolite profiling of methanolic extracts from the medicinal and aromatic species *Mentha pulegium* and *Origanum majorana*. Phytochem Anal..

[18] Barkociová M., Tóth J., Sutor K., Drobnicka N., Wybraniec S., Dudík B., Bilková A., Czigle S., Braca A., De Leo M. (2021). Betalains in edible fruits of three cactaceae taxa-*Epiphyllum, Hylocereus*, and *Opuntia*-their LC-MS/MS and FTIR identification and biological activities. Plants.

[19] Gök H.N., Luca S.V., Ay S.T., Komsta Ł., Salmas R.E., Orhan I.E., Skalicka-Woźniak K. (2022). Profiling the annual change of the neurobiological and antioxidant effects of five *Origanum* species in correlation with their phytochemical composition. Food Chem..

[20] Quirantes-Piné R., Arráez-Román D., Segura-Carretero A. (2010). Fernández-Gutiérrez, A. Characterization of phenolic and other polar compounds in a lemon verbena extract by capillary electrophoresis-electrospray ionization-mass spectrometry. J. Sep. Sci..

[21] Zengin G., Ferrante C., Orlando G., Zheleva-Dimitrova D., Gevrenova R., Recinella L., Chiavaroli A., Leone A., Brunetti L., Aumeeruddy M.Z. (2019). Chemical profiling and pharmaco-toxicological activity of *Origanum sipyleum* extracts: Exploring for novel sources for potential therapeutic agents. J. Food Biochem..

[22] Farag M.A., Shakour Z.T.A. (2019). Metabolomics driven analysis of 11 *Portulaca* leaf taxa as analysed via UPLCESI- MS/MS and chemometrics. Phytochemistry.

[23] Nastić N., Borrás-Linares I., Lozano-Sánchez J., Švarc-Gajić J., Segura-Carretero A. (2020). Comparative assessment of phytochemical profiles of comfrey (*Symphytum officinale* L.) root extracts obtained by different extraction techniques. Molecules.

[24] Flores P., Hellín P., Fenoll J. (2012). Determination of organic acids in fruits and vegetables by liquid chromatography with tandem-mass spectrometry. Food Chem..

[25] Hossain M.B., Rai D.K., Brunton N.P., Martin-Diana A.B., Barry-Ryan C. (2010). Characterization of phenolic composition in *Lamiaceae* spices by LC-ESI-MS/MS. J. Agric. Food Chem..

[26] Guan J., Wang L., Jin J., Chang S., Xiao X., Feng B., Zhu H. (2019). Simultaneous determination of calycosin-7-*O-β-D*-glucoside,cinnamic acid, paeoniflorin and albiflorin in rat plasma by UHPLC-MS/MS and its application to a pharmacokinetic study of *Huangqi guizhi* Wuwu decoction. J. Pharm. Biomed. Anal..

[27] Maurya N.K., Arya P. (2018). Amaranthus grain nutritional benefits: A review. J. Pharmacogn. Phytochem..

[28] Stintzing F.C., Kammerer D., Schieber A., Adama H., Nacoulma O.G., Carle R. (2004). Betacyanins and phenolic compounds from *Amaranthus spinosus* L. and *Boerhavia erecta* L. Z. Naturforsch. C.

[29] Martinez-Lopez A., Millan-Linares M.C. (2020). Rodriguez-Martin, N.M.; Millan, F.; Montserrat-de la Paz, S. Nutraceutical value of Kiwicha (*Amaranthus caudatus* L.). J. Funct. Foods..

[30] Popa I., Solgadi A., Pin D., Watson A.L., Haftek M., Portoukalian J. (2021). The linoleic acid content of the stratum corneum of ichthyotic golden retriever dogs is reduced as compared to healthy dogs and a significant part is oxidized in both free and esterified forms. Metabolites.

[31] Friedrich W. (1987). Handbuch Der Vitamine C.

[32] Rahimi P., Abedimanesh S., Mesbah-Namin S.A., Ostadrahimi A. (2019). Betalains, the nature-inspired pigments, in health and diseases. Crit. Rev. Food Sci. Nutr..

[33] Nemzer B., Pietrzkowski Z., Spórna A., Stalica P., Thresher W., Michałowski T., Wybraniec S. (2011). Betalainic and nutritional profiles of pigment-enriched red beet root (*Beta vulgaris* L.) dried extracts. Food Chem..

[34] Zhang B., Xia T., Duan W., Zhang Z., Li Y., Fang B., Xia M., Wang M. (2019). Effects of organic acids, amino acids and phenolic compounds on antioxidant characteristic of *Zhenjiang* aromatic vinegar. Molecules.

[35] Orabi S.A., Hussein M.M., Abd El-Motty E.Z., El-Faham S.Y. (2018). Effect of alpha-tochopherol and glutamic acid on total phenols, antioxidant activity, yield and fruit properties of mango trees. Science.

[36] Gülçin I. (2007). Comparison of in vitro antioxidant and antiradical activities of *L*-tyrosine and *L*-dopa. Amino Acids.

[37] Hwang H.S., Winkler-Moser J.K., Doll K.M., Gadgil M., Liu S.X. (2019). Factors affecting antioxidant activity of amino acids in soybean oil at frying temperatures. Eur. J. Lipid Sci. Technol..

[38] Zhang B., Deng Z., Tang Y., Chen P., Liu R., Ramdath D.D., Liu Q., Hernandez M., Tsao R. (2014). Fatty acid, carotenoid and tocopherol compositions of 20 canadian lentil cultivars and synergistic contribution to antioxidant activities. Food Chem..

[39] Li L., Tsao R., Yang R., Kramer J.K., Hernandez M. (2007). Fatty acid profiles, tocopherol contents, and antioxidant activities of Heartnut (*Juglans ailanthifolia* var. Cordiformis) and Persian walnut (*Juglans regia* L.). J. Agric. Food Chem..

[40] Grela E.R., Samolińska W., Kiczorowska B., Klebaniuk R., Kiczorowski P. (2017). Content of minerals and fatty acids and their correlation with phytochemical compounds and antioxidant activity of *Leguminous* seeds. Biol. Trace Elem. Res..

[41] Abdoulaye T., Claude K.A.L., Constant A.A.R., Faustin K.A., Marcelline A.N.D., Barthélémy A.K. (2018). Antibacterial and acute toxicity studies of culinary leaves from *Adansonia digitata* L. (Malvaceae) and *Amaranthus cruentus* L. (Amaranthaceae) growing in Côte d’Ivoire. European J. Biotechnol. Biosci..

[42] Marsot A., Boulamery A., Bruguerolle B., Simon N. (2012). Vancomycin. Clin. Pharmacokinet..

[43] Davis R., Markham A., Balfour J.A. (1996). Ciprofloxacin. Drugs.

[44] Goa K.L., Barradell L.B. (1995). Fluconazole. Drugs.

[45] EUCAST (2003). European Committee for Antimicrobial Susceptibility Testing (EUCAST) of the European Society of Clinical Microbiology and Infectious Diseases (ESCMID): Determination of Minimum Inhibitory Concentrations (MICs) of Antibacterial Agents by Broth Dilution. Clin. Microbiol. Inf. Dis..

